# Dynamics of Cardiac Autonomic Responses During Hemodialysis Measured by Heart Rate Variability and Skin Sympathetic Nerve Activity: The Impact of Interdialytic Weight Gain

**DOI:** 10.3389/fphys.2022.890536

**Published:** 2022-05-16

**Authors:** Yike Zhang, Jing Wang, Yantao Xing, Chang Cui, Hongyi Cheng, Zhenye Chen, Hongwu Chen, Chengyu Liu, Ningning Wang, Minglong Chen

**Affiliations:** ^1^ Division of Cardiology, Jiangsu Province Hospital, The First Affiliated Hospital of Nanjing Medical University, Nanjing, China; ^2^ Department of Nephrology, Jiangsu Province Hospital, The First Affiliated Hospital of Nanjing Medical University, Nanjing, China; ^3^ School of Instrument Science and Engineering, Southeast University, Nanjing, China

**Keywords:** hemodialysis, end-stage kidney disease, skin sympathetic nerve activity, heart rate variability, autonomic nervous system, interdialytic weight gain (IDWG)

## Abstract

**Background:** Autonomic nervous regulation plays a critical role in end-stage kidney disease (ESKD) patients with cardiovascular complications. However, studies on autonomic regulation in ESKD patients are limited to heart rate variability (HRV) analysis. Skin sympathetic nerve activity (SKNA), which noninvasively reflects the sympathetic nerve activity, has not been used in ESKD patients.

**Methods:** Seventy-six patients on maintenance hemodialysis (MHD) treatment (a 4-h HD session, three times a week) were enrolled. Utilizing a noninvasive, single-lead, high-frequency recording system, we analyzed the dynamic change in HRV parameters and SKNA during HD. The different characteristics between the subgroups divided based on interdialytic weight gain (IDWG, <3 kg or ≥3 kg) were also demonstrated.

**Results:** After the HD, values for heart rate (75.1 ± 11.3 to 80.3 ± 12.3 bpm, *p* < 0.001) and LF/HF (1.92 ± 1.67 to 2.18 ± 2.17, *p* = 0.013) were significantly higher than baseline. In subgroup analysis, average voltage of skin sympathetic nerve activity (aSKNA) in IDWG ≥3 kg group was lower than the IDWG <3 kg group at the end of MHD (1.06 ± 0.30 *vs* 1.32 ± 0.61 μV, *p* = 0.046). Moreover, there was a linear correlation between mean heart rate (HR) and aSKNA in low IDWG patients (*p* < 0.001), which was not found in high IDWG patients. At the 1-year follow-up, high IDWG patients had a higher incidence of cardiovascular hospitalization (*p* = 0.046).

**Conclusions:** In MHD patients, a gradual activation of sympathetic nerve activity could be measured by HRV and aSKNA. A lower aSKNA at the end of HD and a loss of HR-aSKNA correlation in overhydrated patients were observed. Extensive volume control is promising to improve the autonomic nervous function and clinical outcomes in this population.

## Introduction

End-stage kidney disease (ESKD) has become a rapidly increasing global health burden. Based on the latest epidemiological data from 160 countries worldwide, the average number of new ESKD diagnoses was 144 individuals per million population (Gupta et al., 2021). Most patients with ESKD undergo hemodialysis (HD), and cardiovascular diseases (CVD) remain the leading cause of death (Sud et al., 2014; Modi et al., 2019). The pathophysiology of high cardiovascular mortality among dialysis patients can be attributed to cardiac autonomic dysfunction and uremic cardiomyopathy, which account for almost 48% of deaths (Converse et al., 1992b; Kaur et al., 2017; Saran et al., 2019). In addition, traditional clinical risk factors, such as age, sex, and diabetes mellitus, also contribute to adverse outcomes.

Heart rate variability (HRV) indices reflect autonomic nervous system (ANS) modulation of cardiac activity, particularly the heart rate, through both the sympathetic and parasympathetic nervous systems (Electrophysiology, 1996; Ferrario et al., 2014). Previous studies have revealed that HRV indices could be remarkable predictors for all-cause mortality and cardiovascular deaths (La Rovere et al., 2003; Sessa et al., 2018). Moreover, these parameters were also reported to predict intradialytic hypotension, vascular access survival, and long-term mortality in HD patients (Chang et al., 2016a; Huang et al., 2017; Chang et al., 2020). However, HRV displayed by short periods or 24-h Holter electrocardiograms cannot fully show the real-time nerve activity in patients undergoing HD. Recently, skin sympathetic nerve activity (SKNA) has become a novel noninvasive parameter to reflect real-time cardiac sympathetic nerve activity (Doytchinova et al., 2017; Kusayama et al., 2020). This parameter has been widely used in cardiac arrhythmogenesis, acute myocardial infarction, sleep apnea, and neurologic recovery patients as a predictor of sympathetic tone (Kusayama et al., 2019; He et al., 2020; Chen et al., 2021; Kutkut et al., 2021; Liu et al., 2021). However, in HD patients, the implications of SKNA have not been well elucidated. Furthermore, fluid overload can result in a poor prognosis for HD. Interdialytic weight gain (IDWG), which measures the fluid volume accumulated from the previous session, is associated with higher cardiac events and all-cause mortality (Kalantar-Zadeh et al., 2009; Flythe et al., 2013; Cabrera et al., 2015; Wong et al., 2017). These volume-related outcomes are partly mediated by ANS disturbance. The difference in autonomic modulation has not been thoroughly discussed in patients with different IDWGs.

This study aimed to assess SKNA and HRV simultaneously in patients undergoing HD. We also intended to determine the correlation between overhydration and cardiac autonomic nerve function. This might be a potential mechanism linking overhydration to subsequent adverse prognoses in HD patients.

## Methods

### Patients and Study Design

In this prospective study, adult patients were enrolled from the First Affiliated Hospital of Nanjing Medical University who had been stably undergoing maintenance hemodialysis (>3 months) from August 2020 to November 2020. The exclusion criteria included *1*) less than 18 years old; *2*) had received HD for less than 3 months from initiation; *3*) presence of fever, infection, or pregnancy; *4*) fasting blood glucose over 11.1 mmol/L; *5*) severe hepatic or pulmonary diseases, malignant tumors, or severe mental disorders; *6*) episodes of acute myocardial infarction, stroke, or a major surgical procedure within the past 3 months. *7*) refused the recordings. All the participants provided written informed consent, and all the data were analyzed anonymously.

### Baseline Characteristics

**FIGURE 1:**
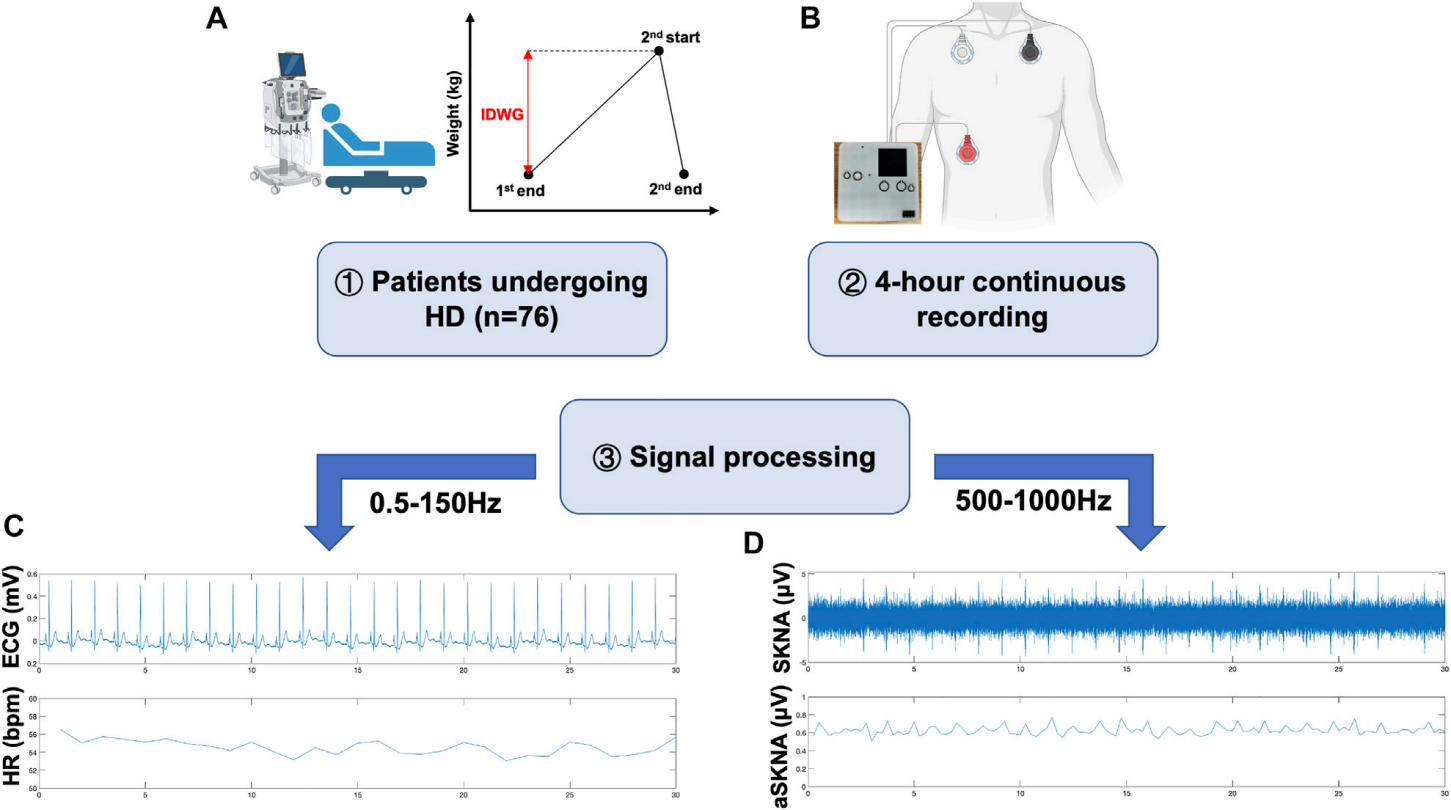
Graphical illustration of this study. **(A)** 76 ESKD patients receiving MHD therapy were included in this study. IDWG refers to the fluid accumulated between two times of HD as illustrated. **(B)** Home-made wearable devices were applied to record high-frequency signals for further analysis. Following previous protocols, electrode patches were placed in the left subclavicular area, right subclavicular area, and right abdomen. **(C**,**D)** Representative workflow of data processing. Raw data was applied to a 500–1000 Hz bandpass filter to get SKNA **(C)** and 0.5–150 Hz bandpass filter to get ECG **(D)**. R-wave peaks were extracted for further HRV analysis using the PhysioNet toolbox.

The following baseline characteristics were collected: demographic information, laboratory tests, comorbidities, and drug usage. Furthermore, IDWG was defined as the change in weight from the end of one dialysis session until the start of the next session (Figure 1A) (Flythe et al., 2013; Hecking et al., 2018). Systolic and diastolic blood pressure (SBP and DBP) were recorded at baseline and tested hourly at 1, 2, 3, and 4 h during HD (five times total). These patients were followed up for 1 year, and hospitalization events were recorded.

### Biochemical Examination

Venous blood samples were taken in the morning after overnight fasting and tested before HD. The testing included assessment of serum creatinine, urea, albumin, blood glucose, total cholesterol, total triglyceride, calcium, phosphorus, iron, ferritin, transferrin, phosphorus, and intact parathyroid hormone (iPTH).

### SKNA and Single-Lead Electrocardiogram (ECG) Recordings

Each patient underwent a continuous 4-h recording during HD. We developed a custom-made device that could simultaneously record SKNA and single-lead ECG signals (Xing et al., 2022), which proved to be consistent with the PowerLab system. Three electrodes (3M™ Red Dot Monitoring Electrode, #2570) were placed in the left subclavian, right subclavian, and right lower abdomen, respectively, as shown in the schematic illustration (Figure 1B). The sampling rate was 4,000 Hz, and the signals were recorded continually during the 4-h HD. The patients were asked to stay supine and avoid unnecessary movement during the recording. Electronic instrument usage, which could produce signal artifacts, was avoided during recordings.

### SKNA Data Processing

SKNA was derived from the raw data signals bandpass filtered at 500–1000 Hz (Kusayama et al., 2020). Next, three quartiles were calculated as Q1, Q2, and Q3. The deviations of Q1 and Q3 were defined as interquartile ranges (IQRs). Mild outliers, data below Q1-1.5 * IQR or above Q3+1.5 * IQR, were excluded. Then, average voltage of skin sympathetic nerve activity (aSKNA) was derived from every 30-min time period (0–30, 30–60, 60–90, 90–120, 120–150, 150–180, 180–210, 210–240 min, eight periods total). The representative signals processed from the raw data are shown in Figure 1C.

### HRV Analysis

HRV analysis was based on the PhysioNet Cardiovascular Signal Toolbox by Vest et al. (Vest et al., 2018). This toolbox includes standardized preprocessing, signal quality indications (for noise segment removal), and abnormal rhythm identification. Beat-to-beat RR intervals were extracted and subjected to time domain analysis, frequency domain analysis, and nonlinear analysis. Lomb–Scargle periodogram was used as the default method for frequency analysis. The averages of 5-min HRV indices every 30 min (0–30, 30–60, 60–90, 90–120, 120–150, 150–180, 180–210, 210–240 min, eight periods total) were calculated during the 4-h HD.

The time domain analysis included mean RR intervals, the standard deviation of all sinus RR intervals (SDNN), the square root of the mean square of differences between adjacent normal-to-normal intervals (RMSSD), acceleration capacity (AC), and deceleration capacity (DC). The frequency domain analysis consisted of total power (TP), high-frequency power (HF), low-frequency power (LF), and the ratio of low to high-frequency power (LF/HF). The nonlinear analysis consisted of SD1, SD2, SD1/SD2, and sample entropy (SampEn).

### Statistical Analysis

The Kolmogorov-Smirnov test was performed for normality. Continuous variables were presented as the mean ± standard deviation (SD) if normally distributed or the median with IQRs if the values were not normally distributed. Categorical variables were expressed as percentages or ratios. Between-group differences were compared by Student’s t test, Mann-Whitney *U* test, chi-square test, or Fisher’s exact test, as appropriate. An independent Student’s t test was performed to compare the HRV and SKNA parameters between two subgroups. Changes of HRV or SKNA parameters in each period were analyzed by one-way repeated measures analysis of variance (ANOVA) adjusted by Bonferroni’s multiple comparisons test (*vs* the baseline). The linear correlation was tested based on Fisher’s Z-transformation. The Chi-square test was used to compare 1-year event rate between groups. Yates’ correction for continuity was performed when at least one cell of the 2*2 table had an expected count between 1 and 5. A *p* value less than 0.05 was considered statistically significant for a two-tailed test. Statistical analyses were performed using SPSS (version 25.0, IBM), Prism 8 (version 8.2.1, GraphPad Software), and MATLAB (R2021a).

## Results

### Subjects

Seventy-six patients were included in this observational study and their baseline characteristics are detailed in Table 1. The patients were divided into two groups: 25 patients with high IDWG (≥3 kg, Group A) and 51 patients with low IDWG (<3 kg, Group B).

**TABLE 1:** Baseline characteristics of HD patients.

	All *N* = 76	Group A (IDWG≥3 kg) *N* = 25	Group B (IDWG<3 kg) *N* = 51	*p*-value
**Demographic Information**				
Age, years	62.0 [48.3–69]	51.0 [44.5–60.5]	66.0 [50.0–71.0]	**0.003**
Gender (male)	48 (63.2%)	18 (72.0%)	30 (58.8%)	0.263
Dialysis vintage,years	3.0 [1.25–5.75]	3.0 [2.0–5.5]	3.0 [1.0–6.0]	0.666
Dry weight, kg	63.9 [57.9–70.1]	66.5 [62.4–76.1]	60.9 [53.4–68.6]	**0.019**
BMI, kg/m^2^	23.1 ± 3.9	24.1 ± 3.6	22.6 ± 4.0	0.116
IDWG, kg	2.5 ± 1.0	3.6 ± 0.6	1.9 ± 0.6	**<0.001**
IDWG/Dry Weight, %	3.9 ± 1.4	5.2 ± 0.6	3.2 ± 1.1	**<0.001**
SBP, mmHg	143.4 ± 20.1	143.8 ± 22.5	143.2 ± 19.1	0.900
DBP, mmHg	77.6 ± 12.2	77.6 ± 11.8	77.5 ± 12.5	0.986
**Laboratory test**				
Albumin, g/L	39.3 ± 4.2	38.3 ± 3.2	39.8 ± 4.5	0.142
Scr, μmol/L	800.8 [683.0–1065.6]	910.0 [690.3–1185.8]	771.6 [678.7–1018.3]	0.116
BUN, mmol/L	25.4 ± 6.5	27.3 ± 6.0	24.5 ± 6.5	0.072
TC, mmol/L	4.4 ± 1.2	4.1 ± 1.0	4.5 ± 1.3	0.298
TG, mmol/L	1.9 [1.4–2.6]	1.8 [1.2–2.4]	1.94 [1.41–2.69]	0.370
Calcium, mmol/L	2.3 ± 0.2	2.3 ± 0.2	2.3 ± 0.2	0.083
Phosphate, mmol/L	1.8 ± 0.4	1.9 ± 0.4	1.8 ± 0.5	0.118
Iron, μmol/L	12.9 ± 6.3	11.5 ± 4.8	13.5 ± 6.9	0.197
Ferritin, μg/L	144.6 [78.6–434.5]	98.7 [63.1–175.7]	214.1 [99.0–536.4]	**0.007**
Transferrin, g/L	1.7 ± 0.3	1.8 ± 0.4	1.7 ± 0.3	0.293
iPTH, pg/ml	225.1 [87.5–484.7]	345.8 [88.6–790.3]	187.3 [85.1–430.5]	0.146
**Comorbidities**				
Diabetes	28 (36.8%)	11 (44.0%)	17 (33.3%)	0.365
Hypertension	60 (78.9%)	22 (88.0%)	38 (74.5%)	0.175
Stroke	11 (14.5%)	4 (16.0%)	7 (13.7%)	1.000
Pulmonary infection	3 (3.9%)	1 (4.0%)	2 (3.9%)	1.000
Vasculitis	1 (1.3%)	0 (0.0%)	1 (2.0%)	1.000
SLE	1 (1.3%)	1 (4.0%)	0 (0.0%)	0.329
CAD	15 (19.7%)	5 (20.0%)	10 (19.6%)	1.000
Heart failure	8 (10.5%)	4 (16.0%)	4 (7.8%)	0.490
**Drugs**				
β-blocker	33 (43.4%)	13 (52.0%)	20 (39.2%)	0.291
ACEI/ARB	17 (22.4%)	4 (16.0%)	13 (25.5%)	0.351

BMI, body mass index; IDWG, interdialytic weight gain; SBP, systolic blood pressure at baseline; DBP, diastolic blood pressure at baseline; Scr, serum creatinine; BUN, blood urea nitrogen; TC, total cholesterol; TG, triglyceride; iPTH, intact parathyroid hormone; SLE, systemic lupus erythematosus; CAD, coronary artery disease; ACEI/ARB, angiotensin-converting enzyme inhibitors/angiotensin receptor blockers.

The patients in Group A were younger than those in Group B (51.0 [44.5, 60.5] *vs* 66.0 [50.0, 71.0] years, *p* = 0.003), with higher dry weight (66.5 [62.4, 76.1] *vs* 60.9 [53.4, 68.6] kg, *p* = 0.019) and lower serum ferritin (98.7 [63.1, 175.7] *vs* 214.1 [99.0, 536.4] μg/L, *p* = 0.007). There were no significant differences in other baseline characteristics between the two groups.

### Sympathetic Activation Revealed by HRV and SKNA Parameters During 4-h HD

**FIGURE 2:**
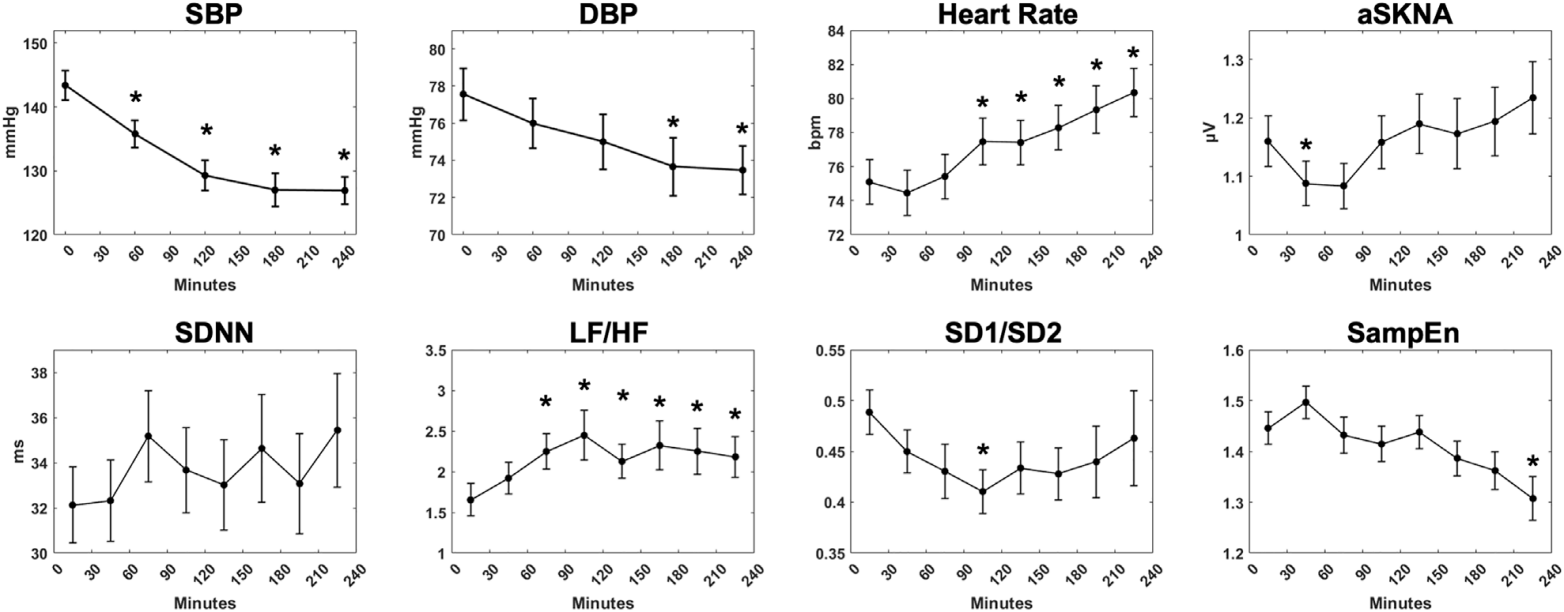
The overall trend of physiological measurements during 4-h hemodialysis. Systolic blood pressure, diastolic blood pressure, mean heart rate, average skin sympathetic nerve activity, and representative HRV indices (SDNN, LF/HF, SD1/SD2, SampEn) were demonstrated. “*” stands for statistically significant (*p* < 0.05) by one-way repeated measures ANOVA adjusted by Bonferroni’s multiple comparisons test (*vs* the baseline). Error bars stand for the standard error of the mean (SEM).

Physiological parameters of 76 patients during 4-h HD are shown in Figure 2. During 4-h HD, blood pressure was measured at the initiation and during HD every hour. SBP (143.4 ± 20.1 to 126.9 ± 18.9 mmHg, *p* < 0.001) and DBP (77.6 ± 12.2 to 73.5 ± 11.3 mmHg, *p* = 0.003) decreased gradually as the fluid was removed. The mean heart rate raised from 75.1 ± 11.3 bpm in 0–30 min to 80.3 ± 12.3 bpm in 210–240 min (*p* < 0.001). The aSKNA showed a slow upward trend over time but did not show statistical significance. In time domain HRV indices, SDNN tended to fluctuate. In contrast, the frequency domain parameter LF/HF showed an upward trend during HD (1.92 ± 1.67 to 2.18 ± 2.17, *p* = 0.013). The nonlinear parameter sample entropy, which reflects the complexity of signals, showed a downward trend from 1.45 ± 0.28 in 0–30 min to 1.31 ± 0.37 in 210–240 min (*p* = 0.038).

### Changes in SKNA and aSKNA-HR Correlations During HD

**FIGURE 3:**
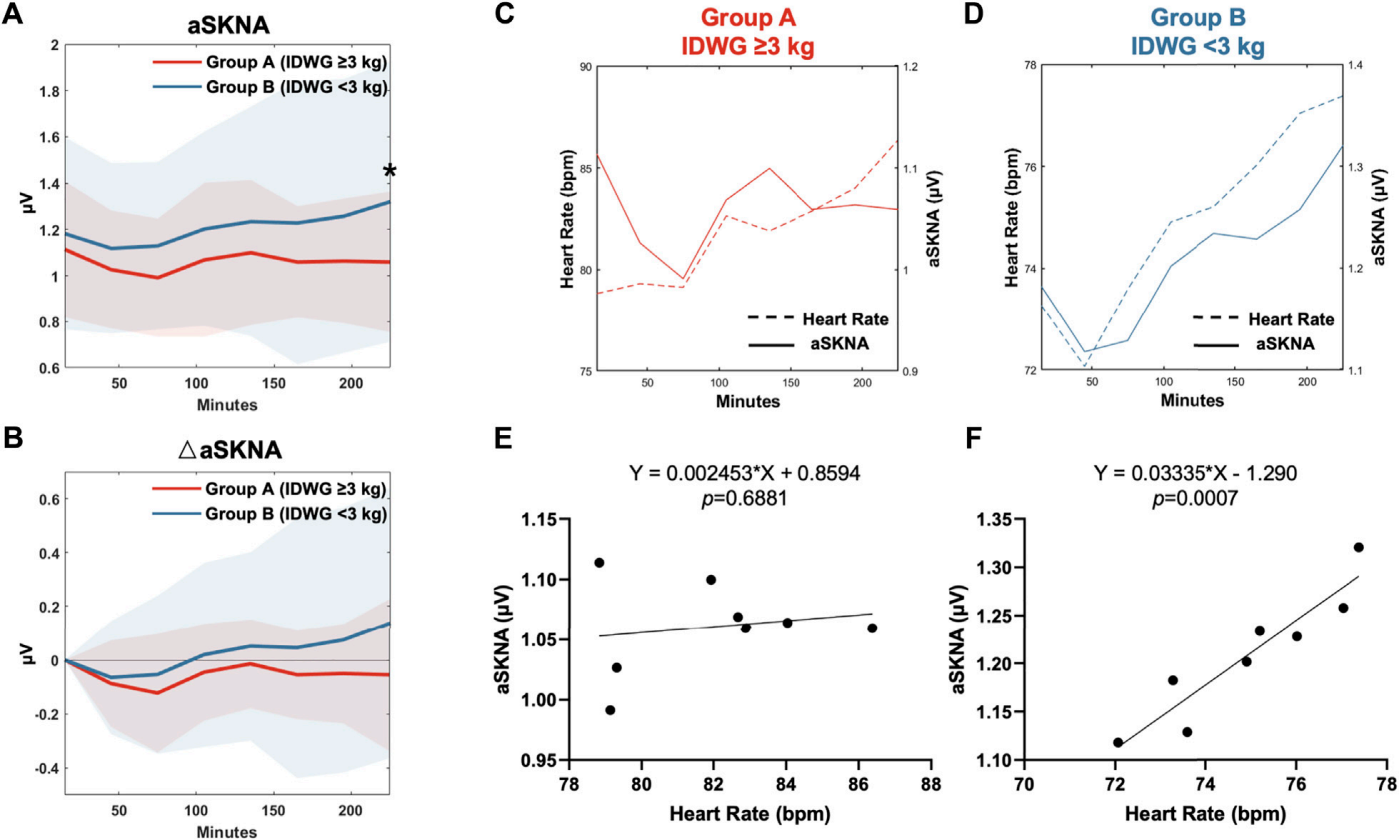
HR-aSKNA correlation between Group A (IDWG≥3 kg) and Group B (<3 kg). **(A**,**B)** Subgroup analysis showed aSKNA **(A)** and the difference of aSKNA to the baseline in 0–30 min (△aSKNA) **(B)**. The aSKNA in 210–240 min was significantly higher in Group B (IDWG <3 kg). “*” stands for statistically significant (*p* < 0.05) by Student’s t test between two subgroups. **(C**,**D)** The relationship of HR and aSKNA in Group A **(C)** or Group B **(D)** was demonstrated in eight periods (0–30, 30–60, 60–90, 90–120, 120–150, 150–180, 180–210, 210–240 min). **(E**,**F)** There was no HR-aSKNA correlation observed in patients in Group A (**E**, *p* = 0.688). Instead, a linear correlation was found in Group B (**F**, *p* < 0.001).

In the subgroup analysis, the mean heart rate in Group A at baseline and during HD were both higher than those in Group B. The aSKNA at baseline was not significantly different between the two groups (1.11 ± 0.29 *vs* 1.18 ± 0.42 μV, *p* = 0.463). During HD, the aSKNA gradually increased (Figure 3A). At the end of HD, aSKNA was significantly higher in Group B (1.06 ± 0.30 *vs* 1.32 ± 0.61 μV, *p* = 0.046). The extent of aSKNA increase in Group B was more prominent than that in Group A but did not reach statistical significance (Figure 3B).

Furthermore, we analyzed the relationship between aSKNA and mean HR during the eight periods of HD in Group A (Figure 3C) and Group B (Figure 3D). No significant relationship between changes in aSKNA and HR was observed in Group A (*p* = 0.688, *R* ^2^ = 0.871) (Figure 3E). In contrast, in Group B, aSKNA and HR increased in pace with each other and were significantly correlated (*p* < 0.001, *R* ^2^ = 0.029) (Figure 3F).

### Effect of Fluid Overload on HRV Parameters in HD Patients

**FIGURE 4:**
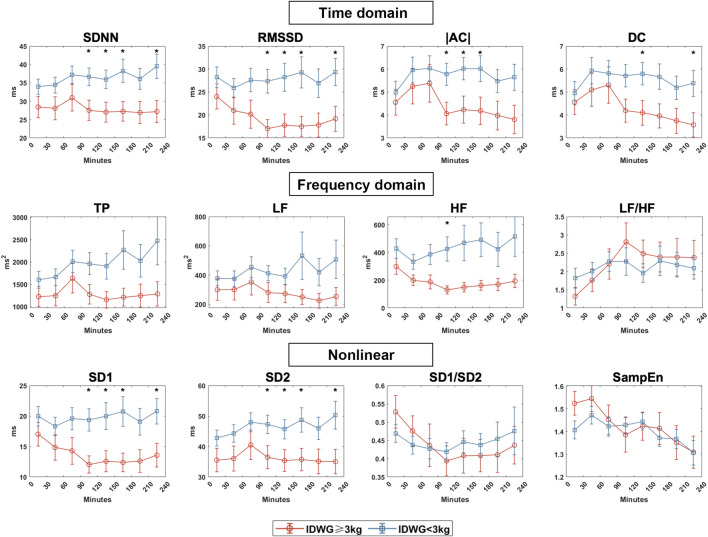
HRV indices between Group A (IDWG≥3 kg) and Group B (IDWG<3 kg). Representative time domain (SDNN, RMSSD, |AC|, DC), frequency domain (TP, LF, HF, LF/HF), and nonlinear (SD1, SD2, SD1/SD2, SampEn) measurement of HRV between subgroups. Red and blue lines stand for Group A (IDWG≥3 kg) and Group B (IDWG<3 kg), respectively. “*” stands for statistically significant (*p* < 0.05) by Student’s t test between two subgroups. Error bars stand for the standard error of the mean (SEM).

**FIGURE 5:**
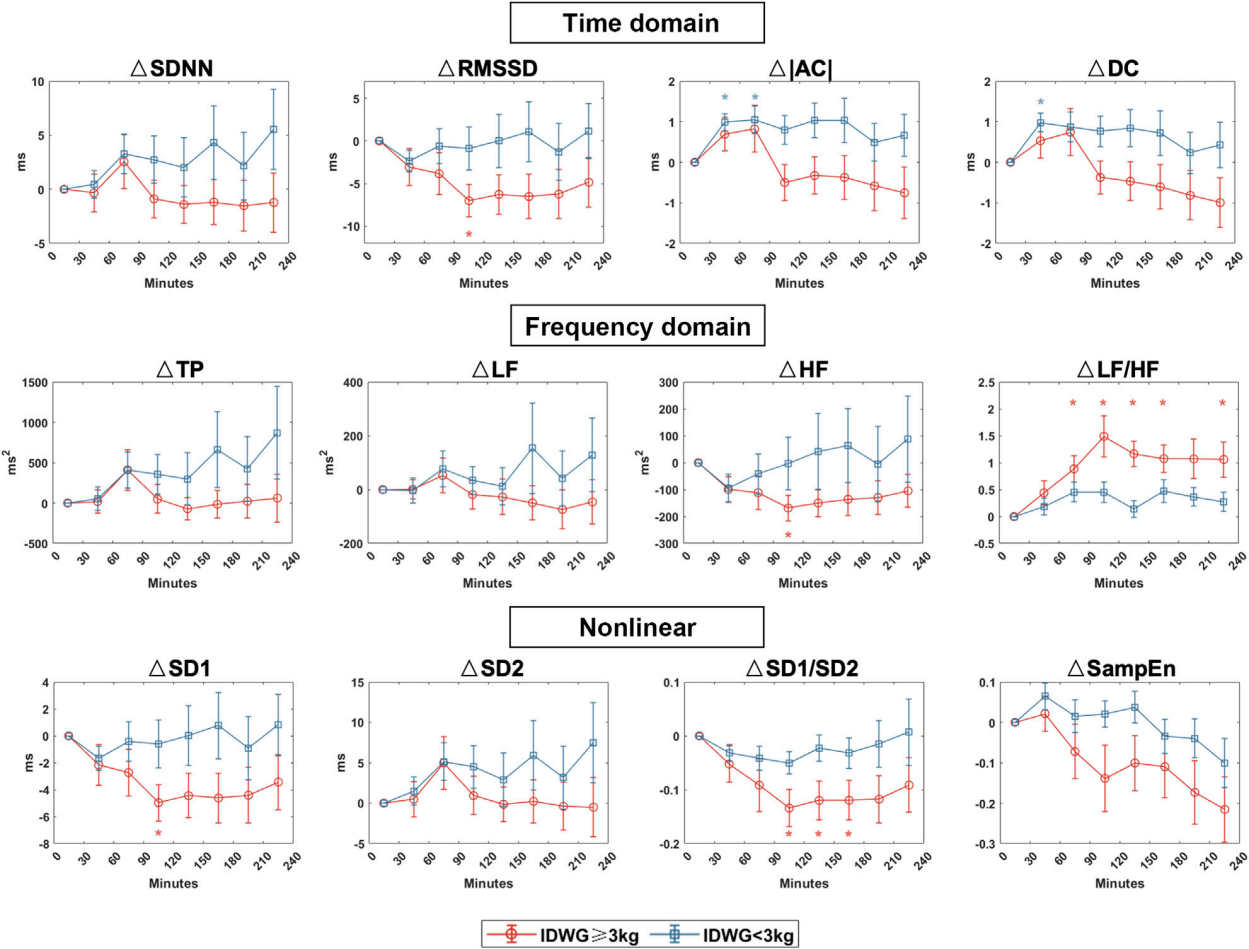
HRV dynamic changes in Group A (IDWG≥3 kg) and Group B (IDWG<3 kg). Changes in the time domain (SDNN, RMSSD, |AC|, DC), frequency domain (TP, LF, HF, LF/HF), and nonlinear (SD1, SD2, SD1/SD2, SampEn) measurement of HRV compared with 0–30 min in two subgroups. Red and blue lines or “*” stand for IDWG≥3 kg and IDWG<3 kg, respectively. “△” stands for the difference compared to the baseline. “*” stands for statistically significant (*p* < 0.05) by one-way repeated measures ANOVA adjusted by Bonferroni’s multiple comparisons tests (*vs* the baseline in 0–30 min). Error bars stand for the standard error of the mean (SEM).

We compared the autonomic nerve activities between groups based on traditional HRV parameters during HD in eight periods (0–30, 30–60, 60–90, 90–120, 120–150, 150–180, 180–210, 210–240 min) (Figure 4). For time domain HRV indices, SDNN and RMSSD were significantly lower in Group A than in Group B (90–120, 120–150, 150–180, 210–240 min). For frequency domain indices, LF and HF were higher in Group B than in Group A but with no statistical significance. Both groups showed an upward change in LF/HF, but the rise was more drastic in Group A. For nonlinear indices, SD1 and SD2 were significantly higher in Group B than in Group A (90–120, 120–150, 150–180, 210–240 min). SD1/SD2 did not vary significantly but was more unstable in Group A. There was no significant difference in sample entropy between the two subgroups in each period. To demonstrate the changes in all of the HRV parameters, we also showed the difference of each parameter to the baseline in 0–30 min (Figure 5).

### One-Year Follow-Up of Hospitalization Between Two Subgroups in HD Patients

At the 1-year follow-up, 37 hospital admissions were documented (Table 2). In Group A, 11/25 patients had at least one admission during the year compared with 13/51 patients in Group B (*p* = 0.103). Cardiovascular admissions were 7/25 in Group A and 4/51 in Group B (*p* = 0.046). The causes of cardiovascular hospitalizations are detailed in Table 2, including coronary artery diseases (CAD), transient ischemic attack (TIA), hypertension, arrhythmia, and heart failure. One patient died because of central nervous system infection.

**TABLE 2:** One-year follow-up of the hospital admissions.

	All *N* = 76	Group A (IDWG≥3 kg) *N* = 25	Group B (IDWG<3 kg) *N* = 51	*p*-value
**1-year hospital admissions**				0.103^a^
T0	52	14	38	—
T≥1	24	11	13	—
**1-year cardiovascular admissions**				**0.046** ^a^
T0	65	18	47	—
T≥1	11	7	4	—
**Causes of cardiovascular admissions**				
Total	14	10	4	—
CAD	8	7	1	—
Stroke or TIA	3	0	3	—
Others^b^	3	3	0	—

CAD, Coronary artery diseases; TIA, transient ischemic attack.

a *p*-values for two-sided Pearson’s chi-square tests and Yates’ continuity correction if at least one cell has an expected count between 1 and 5.

b Hypertension, arrhythmia, or heart failure.

## Discussion

The major findings in this study are as follows: *1*) We first demonstrated the dynamics of sympathetic modulation in HD patients with a noninvasive approach integrating HRV and SKNA; *2*) The two subgroups divided by IDWG of 3 kg showed different modes of sympathetic modulation during the 4-h HD. Those with low IDWGs showed a better HR-aSKNA correlation; *3*) High IDWG patients had a higher incidence of 1-year cardiovascular hospitalization. These findings could offer new insight into the impact of volume overload on the ANS in HD patients.

### Volume-Induced ANS Response Revealed by HRV Indices in HD Patients

HRV reflects the balanced state of sympathetic and parasympathetic nerve activities. It refers to the response of cardiac postjunctional sinoatrial receptors to oscillations in sympathetic and vagal discharge rather than the direct recordings of nerve discharge (Electrophysiology, 1996). During HD, a large volume of fluid is removed quickly from the body, inducing elevated sympathetic activity and baroreflex activation, thus leading to an increased heart rate. Previous studies demonstrated that time domain parameters such as SDNN and frequency domain parameters such as LF and LF/HF increased during HD in most cases (Rubinger et al., 2013; Chang et al., 2016b; Chang et al., 2020), which are close to our results. The study population should also be noted when comparing HRV with other studies. Our work included MHD patients with a median dialysis vintage of 3 years and the use of β-blockers was 33/76 (43.4%), which could decrease sympathetic activity and affect the results of HRV. We think that the hemodynamic changes caused by ultrafiltration have a greater impact on ANS than that of drugs during 4-h HD. In addition, the signals and analysis in our work covered the whole length of the HD process, which could better demonstrate the changes of HRV parameters and dynamics of cardiac autonomic response.

### Clinical Findings with SKNA Assessment

HRV measurement is indirect and cannot truly represent the real-time physiological state of cardiac sympathetic nerve activity. The results of short-term HRV were also sometimes irreproducible (Deussing et al., 2020). HRV cannot be applied in patients with abnormal heart rhythm, such as atrial fibrillation or repetitive premature ventricular contractions, which are common in HD patients. Recently, the technique of SKNA recording, which is a proper complement to traditional HRV analysis, has provided us with new avenues for study. SKNA signals can be recorded noninvasively by standard ECG patch electrodes and have a linear correlation with the direct recording of stellate ganglion nerve activity (SGNA) (Jiang et al., 2015). In a work by Converse et al. (Converse et al., 1992a), sympathetic activity was documented by microneurography, which can directly record efferent postganglionic muscle sympathetic nerve activity (MSNA). In our study, the elevation of SKNA during HD was also observed using a noninvasive approach. Furthermore, we found a loss of HR-aSKNA correlation in the high IDWG subgroup. Low SKNA or a low correlation between SKNA and heart rate was previously associated with worse neurologic recovery outcomes in cardiac arrest patients undergoing targeted temperature management (Kutkut et al., 2021).

### Fluid Overload, Autonomic System Imbalance, and Adverse Cardiac Outcomes

Fluid accumulation in HD patients is reportedly associated with higher mortality and cardiovascular events. In a retrospective study including 39,256 dialysis patients, absolute IDWG >3 kg was associated with a higher hazard ratio of major adverse cardiovascular events (MACE) and heart failure (Cabrera et al., 2015). In another study of 34,107 HD patients, IDWG >4 kg was associated with a higher risk of cardiovascular death (hazard ratio 1.12–1.39) (Kalantar-Zadeh et al., 2009). Therefore, the hydration state is significant to homeostasis and is tightly linked to the outcomes of HD patients. As an intermediate factor, cardiovascular events contribute to the correlation between IDWG and mortality.

The volume fluctuation can easily affect the cardiac autonomic nervous system. Ferrario et al. reported different HRV parameters in an observational study of 69 HD patients (Ferrario et al., 2014). Patients were divided based on a 2.5 L fluid overload cutoff measured by body impedance. Indices such as the standard deviation of the averages of NN intervals in all 5-min segments of the recording (SDANN), VLF, Lempel Ziv complexity (LZC), and HF% were significantly correlated with fluid overload. In contrast, LF% and LF/HF were inversely correlated with hydration status. Reduced HRV and a reduced sympathetic response to HD can be found in fluid overload patients. In another study, 80 HD patients were divided into three tertiles based on fluid overload normalized over the extracellular water (FO/ECW%) (Ferrario et al., 2015a). In the group with the highest hydration, LF showed no change, but in the lowest hydration group, LF increased during HD.

Our findings showed that HD treatment could impact the HRV indices. The balance between sympathetic and parasympathetic nervous system activity shifted toward the sympathetic nerve hyperactivity, demonstrated by a gradual increase of LF/HF. In a subgroup comparison using a cutoff value of IDWG = 3 kg, we found that the change of time domain parameters (SDNN, RMSSD, AC, and DC) was more noticeable in the low-IDWG group, whereas the high IDWG group remained stable. As demonstrated by the frequency domain parameters, the autonomic modulation of high-IDWG patients was imbalanced, with overactivation of the sympathetic nervous system. In nonlinear parameters, SD1/SD2 declined in the high IDWG patients but was stable in those with low fluid overload. Sample entropy went down in both subgroups but did not reach statistical significance. Nonlinear parameters were widely used in recent works to indicate ANS function and predict outcomes in HD patients (Ferrario et al., 2015b; Tsuji et al., 2017; Liu et al., 2020). Further investigation with a larger sample size is required.

Since HRV is not real-time and indicates the combined activity of the sympathetic and parasympathetic nerves, the application of SKNA in our study provided a distinct view of the sympathetic trend in these two groups. In a previous study using SKNA recordings, the patients with obstructive sleep apnea (OSA) had higher aSKNA, implying a higher sympathetic tone (He et al., 2020). We did not find a difference between subgroups in baseline aSKNA, which could be related to the small sample size and confounding factors. However, the response of aSKNA during HD varied between the two subgroups at the end of HD. Due to the sympathetic nerve overactivity, patients with a high volume load had impaired capacity for adjustment, reflected by a relatively constant aSKNA. We also found a high correlation between aSKNA and mean heart rate in the low IDWG group, which indicates that the heart rate fluctuated with the cardiac sympathetic activity. In contrast, this correlation was missing in the high IDWG patients, implying sympathetic dysregulation and worse clinical outcomes.

### Fluid Management in HD Patients

The relationship between the hydration state in MHD patients and outcomes underlines the significance of normohydration. Strategies that reduce fluid load can prevent adverse cardiovascular effects and improve survival among HD patients. In a secondary analysis of the frequent hemodialysis trials (the Daily Trial and the Nocturnal Trial), it was shown that better volume control could beneficially decrease left ventricular mass, which is correlated with mortality and cardiovascular morbidity (Raimann et al., 2016).

Some studies have shown that reducing fluid overload for 3 months with a strict fluid management plan improved HRV indices (Ferrario et al., 2014). In the future, SKNA is promising to become another indicator for autonomic nervous system assessment in MHD patients.

## Limitation

This study was a single-center, prospective, observational study with small sample size. Patients with diabetes or using β-blockers were not excluded, which could affect the autonomic function and HRV or SKNA recordings. Additionally, follow-up data were limited to 1 year and lacking in major clinical events (e.g., all-cause mortality, cardiovascular mortality). Long-term follow-up is warranted to consolidate our findings further. The laboratory tests were performed only at baseline, and body impedance data were unavailable.

## Conclusion

In MHD patients, the dynamics of sympathetic nerve activity could be assessed by HRV and SKNA. We found a lower aSKNA at the end of HD and a loss of HR-aSKNA correlation in overhydrated patients. Extensive volume control is promising to improve autonomic nervous function and clinical outcomes in MHD patients.

## Data Availability

The raw data supporting the conclusions of this article will be made available by the authors, without undue reservation.
